# Supplementary material for: Impact of Maternal Nutritional Supplementation during Pregnancy and Lactation on the Infant Gut or Breastmilk Microbiota: A Systematic Review
Source: Nutrients. 2021 Mar 30;13(4):1137. doi: 10.3390/nu13041137 (PMC8067242; doi:10.3390/nu13041137)
Supplement: Supplementary file 1 [file nutrients-13-01137-s001.pdf]

Supplementary Data

Supplementary Table 1. PRISMA Checklist.

Section/topic	#	Checklist item	Reported on page #
**TITLE**			
Title	1	Identify the report as a systematic review, meta-analysis, or both.	1
**ABSTRACT**			
Structured summary	2	Provide a structured summary including, as applicable: background; objectives; data sources; study eligibility criteria, participants, and interventions; study appraisal and synthesis methods; results; limitations; conclusions and implications of key findings; systematic review registration number.	1
**INTRODUCTION**			
Rationale	3	Describe the rationale for the review in the context of what is already known.	1-2
Objectives	4	Provide an explicit statement of questions being addressed with reference to participants, interventions, comparisons, outcomes, and study design (PICOS).	2
**METHODS**			
Protocol and registration	5	Indicate if a review protocol exists, if and where it can be accessed (e.g., Web address), and, if available, provide registration information including registration number.	3
Eligibility criteria	6	Specify study characteristics (e.g., PICOS, length of follow-up) and report characteristics (e.g., years considered, language, publication status) used as criteria for eligibility, giving rationale.	3
Information sources	7	Describe all information sources (e.g., databases with dates of coverage, contact with study authors to identify additional studies) in the search and date last searched.	3
Search	8	Present full electronic search strategy for at least one database, including any limits used, such that it could be repeated.	3
Study selection	9	State the process for selecting studies (i.e., screening, eligibility, included in systematic review, and, if applicable, included in the meta-analysis).	3
Data collection process	10	Describe method of data extraction from reports (e.g., piloted forms, independently, in duplicate) and any processes for obtaining and confirming data from investigators.	3
Data items	11	List and define all variables for which data were sought (e.g., PICOS, funding sources) and any assumptions and simplifications made.	3

Risk of bias in individual studies	12	Describe methods used for assessing risk of bias of individual studies (including specification of whether this was done at the study or outcome level), and how this information is to be used in any data synthesis.	4
Summary measures	13	State the principal summary measures (e.g., risk ratio, difference in means).	3-4
Synthesis of results	14	Describe the methods of handling data and combining results of studies, if done, including measures of consistency (e.g., I^2) for each meta-analysis.	4

**Section/topic**	**#**	**Checklist item**	**Reported on page #**
Risk of bias across studies	15	Specify any assessment of risk of bias that may affect the cumulative evidence (e.g., publication bias, selective reporting within studies).	4
Additional analyses	16	Describe methods of additional analyses (e.g., sensitivity or subgroup analyses, meta-regression), if done, indicating which were pre-specified.	3-4
**RESULTS**			
Study selection	17	Give numbers of studies screened, assessed for eligibility, and included in the review, with reasons for exclusions at each stage, ideally with a flow diagram.	4
Study characteristics	18	For each study, present characteristics for which data were extracted (e.g., study size, PICOS, follow-up period) and provide the citations.	7-8
Risk of bias within studies	19	Present data on risk of bias of each study and, if available, any outcome level assessment (see item 12).	7-8
Results of individual studies	20	For all outcomes considered (benefits or harms), present, for each study: (a) simple summary data for each intervention group (b) effect estimates and confidence intervals, ideally with a forest plot.	9-12
Synthesis of results	21	Present results of each meta-analysis done, including confidence intervals and measures of consistency.	15
Risk of bias across studies	22	Present results of any assessment of risk of bias across studies (see Item 15).	6 (Suppleme ntary material)
Additional analysis	23	Give results of additional analyses, if done (e.g., sensitivity or subgroup analyses, meta-regression [see Item 16]).	16-17
**DISCUSSION**			

Summary of evidence	24	Summarize the main findings including the strength of evidence for each main outcome; consider their relevance to key groups (e.g., healthcare providers, users, and policy makers).	17-19
Limitations	25	Discuss limitations at study and outcome level (e.g., risk of bias), and at review-level (e.g., incomplete retrieval of identified research, reporting bias).	20
Conclusions	26	Provide a general interpretation of the results in the context of other evidence, and implications for future research.	20
**FUNDING**			
Funding	27	Describe sources of funding for the systematic review and other support (e.g., supply of data); role of funders for the systematic review.	20

Supplementary Material: Search strategy

((maternal OR mother OR antenatal OR postnatal OR perinatal OR breastfeeding OR pregnant woman OR pregnancy OR lactation OR lactating) AND (nutrition OR diet OR supplement* OR vitamin OR mineral OR probiotic OR prebiotic OR lactobacillus OR bifidobacter* OR saccharomyces OR symbiotic OR oil supplement OR DHA OR docosahexaenoic acid OR omega 3 fatty acid supplement OR fatty acid OR omega-6 OR fish oil OR non-omega-3 fatty acid)) AND (((breast milk OR breastmilk OR human milk) AND (microbiome OR microbiota OR microflora OR bacteria OR microbe OR component OR composition)) OR ((infant OR offspring OR neonate OR baby OR babies OR newborn OR new born) AND (gut OR gastrointestinal OR intestine OR meconium OR stool OR faecal OR fecal OR enteric) AND (microbiome OR microbiota OR microflora OR flora OR bacteria OR microbe OR dysbiosis'))).

The following database specific limiters will be applied to our searches:
• PubMed: clinical trial, randomised control trial, observational study
• Embase: clinical trial, randomised control trial and controlled clinical trial
• Web of Science: articles, ‘AND ((randomised control trial OR observational study OR clinical trial)’ will be added to the search strategy

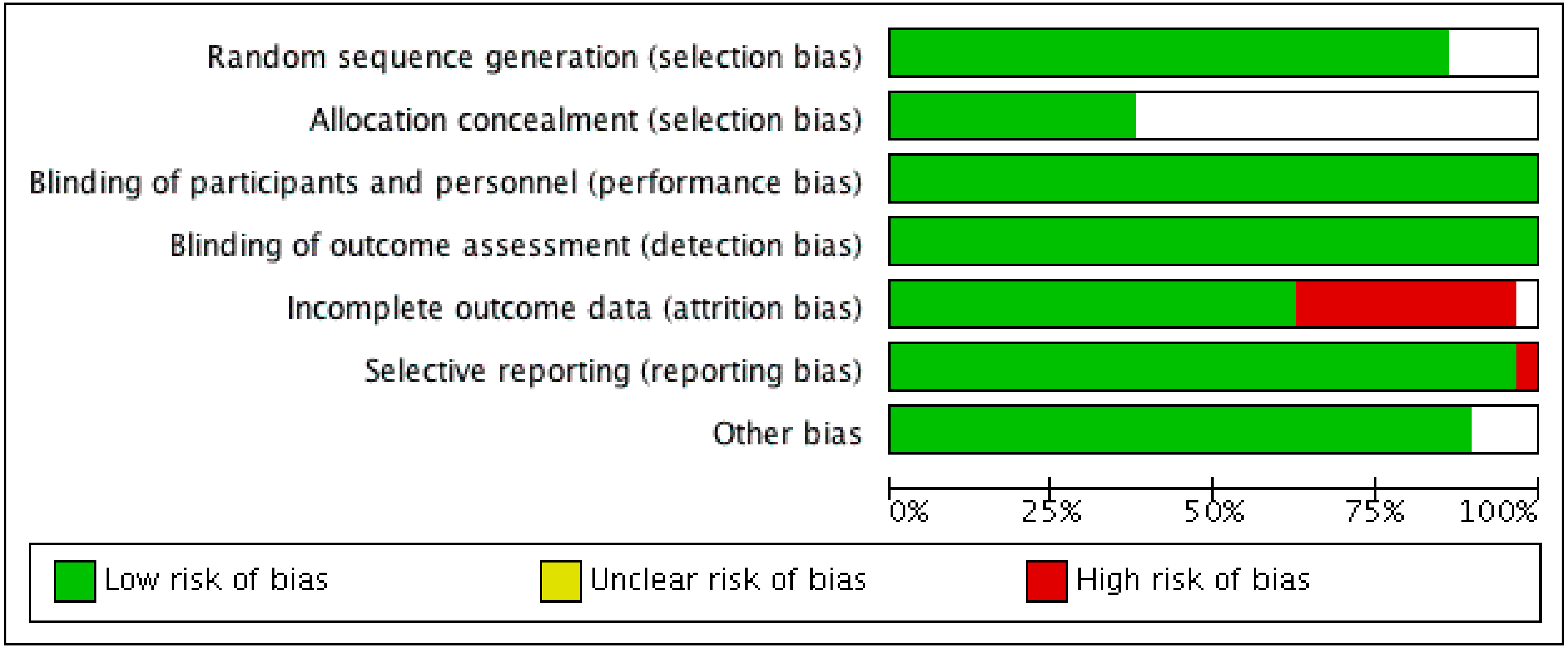

Supplementary Figure 1. Risk of bias graph: judgement of each risk of bias as percentages across all the included studies

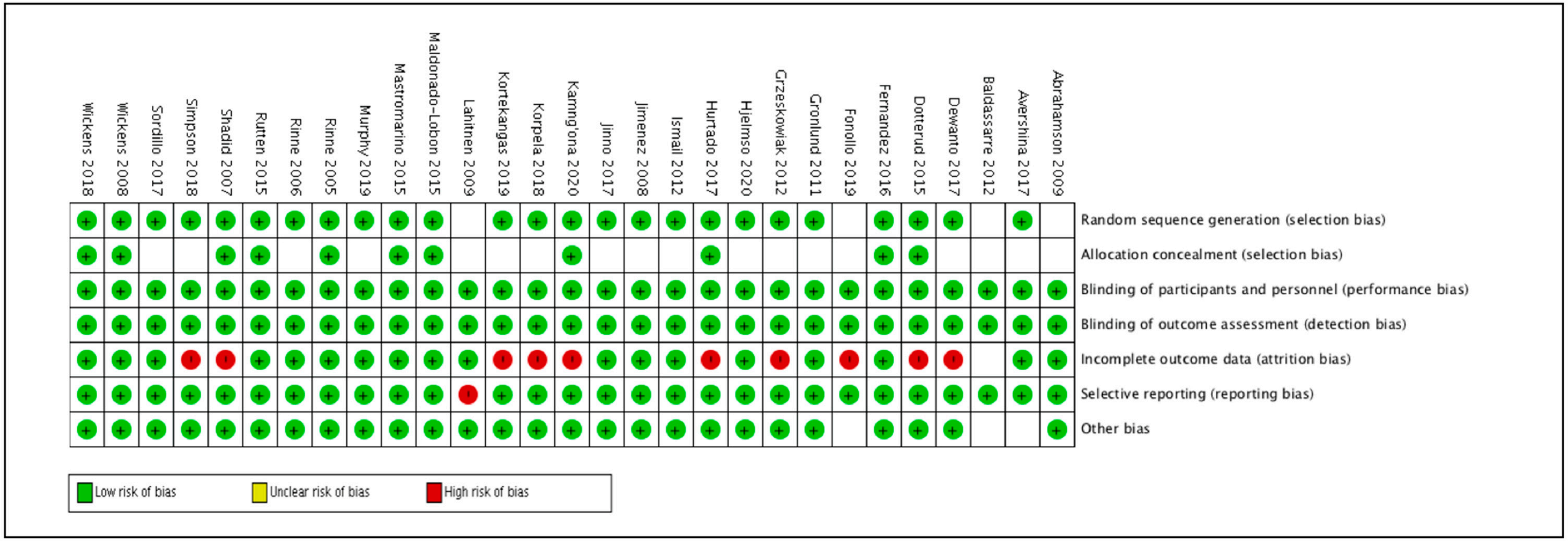

Supplementary Figure 2. Risk of bias summary: judgement of each risk of bias for all included studies

Supplementary Table 2. Methodology of studies included.

Reference	Method of Collection	Method of Assessment
Abrahamsson et al. [24]	Stool samples were collected by parents into sterile plastic containers, they were then immediately placed in the freezer	Gram staining
Avershina et al. [25]	Stool samples frozen -20°C upon defecation and stored at -80°C	RT PCR DNA<br>Primers were designed based on 16srRNA
Baldassarre et al. [20]	Not stated*	Not stated*
Dewanto et al. [21]	Trained person collected the milk sample with an aseptic technique<br>Milk was pumped and then poured into a sterile tube	RT PCR DNA<br>Primers were designed based on 16srRNA
Dotterud et al. [26]	Stool samples were collected from the nappy by the parents and stored at -18°C at home before being transferred to permanent storage at -80°C	RT PCR DNA<br>Primers were designed based on 16srRNA
Fernández et al. [41]	The nipple and mammary areola were cleaned with soap and sterile water, chlorhexidine was applied<br>Breast milk sample was collected in a sterile tube after manual expression using sterile gloves<br>The first drops were discarded to avoid chlorhexidine contamination	Samples spread onto agar plates<br>Colonies identified by matrix-assisted laser desorption/ionization
Fonollá Joya et al. [22]	Not stated*	Not stated*
Grönlund et al. [27]	Stool samples were collected by parents	RT PCR DNA<br>Primers were designed based on 16srRNA
Grześkowiak et al. [28]	Stool samples were collected by parents	RT PCR DNA<br>Primers were designed based on 16srRNA
Hjelmsø et al. [29]	Stool samples were collected either at the research clinic or by the parents at home using detailed instructions<br>Each sample was mixed on arrival with 10% glycerol broth<br>All samples were stored at −80 °C	RT PCR DNA<br>Primers were designed based on 16srRNA
Hurtado et al. [44]	Nipple and mammary areola were cleaned with soap and water and chlorhexidine was applied	Samples spread onto agar plates

	Samples were obtained by manual expression and collected in sterile tubes after discarding the first drops of milk<br>Samples were preserved at -20°C	*Staphylococci, Streptococci*, and *Lactobacilli* counts<br>were measured by quantitative PCR
Ismail et al. [30]	Faecal samples were collected and stored frozen at -80°C	RT PCR DNA<br>Primers were designed based on 16srRNA
Jiménez et al. [42]	The nipple and mammary areola were cleaned with soap and sterile water, and chlorhexidine was applied<br>Breast milk samples were collected in a sterile tube after manual expression using sterile gloves<br>The first drops were discarded to avoid chlorhexidine contamination	Novel multiplex PCR method based on the DNA J genes
Jinno et al. [31]	Stool samples were collected, and mothers were asked to freeze immediately<br>Samples were then kept frozen and stored at -20°C until analysis	RT PCR DNA<br>Primers were designed based on 16srRNA
Kamng'ona et al. [18]	Mothers collected stool samples<br>The tubes were sealed, labelled, and immediately stored in a Ziploc bag on a frozen ice pack in a cooler bag.<br>The samples were transported at -20°C freezer for ≤2 days before frozen at -80°C	RT PCR DNA<br>Primers were designed based on 16srRNA
Korpela et al. [32]	Faecal samples were collected from the infants and stored at −40 °C	RT PCR DNA<br>Primers were designed based on 16srRNA
Kortekangas et al. [19]	Faecal samples were collected from the infant by the parents<br>If a child had diarrhoea, no faecal sample was collected, and the visit was postponed<br>Samples were frozen at -20°C and deep frozen at -80°C	RT PCR DNA<br>Primers were designed based on 16srRNA
Lahtinen et al. [33]	Infant faecal samples and breast milk samples were collected by the mother	RT PCR DNA<br>Primers were designed based on 16srRNA
Maldonado-Lobón et al. [43]	Nipple and mammary areola were cleaned with soap and water and chlorhexidine was applied<br>Samples were obtained by manual expression and collected in sterile tubes after discarding the first drops of milk<br>Samples were preserved at -20°C	RT PCR DNA<br>Primers were designed based on 16srRNA

Mastromarino et al. [45]	Breast milk samples were collected in sterile plastic tubes, using a manual breast-pump, after cleaning the nipples and areola by wiping with a swab soaked in sterile water, and immediately frozen at −80°C	RT PCR DNA<br>Primers were designed based on 16srRNA
Murphy et al. [34]	Parents collected faecal samples and frozen by the in their home freezers at -20 °C<br>Frozen samples were transported on ice to the research centres and stored at -80 °C	Shotgun metagenomic sequencing analysis
Rinne et al. [36]	Faecal specimens were collected from nappies and immediately cooled to 8°C and transported to the hospital within 24 hours to be frozen at -75°C	FISH analysis
Rinne et al. [35]	Faecal specimens were collected from nappies and immediately cooled to 8°C and transported to the hospital within 24 hours to be frozen at -75°C	FISH analysis
Rutten et al. [37]	Stool samples were collected from nappies or caught on a sheet, placed in stool collection vials and immediately frozen by the parents in their home freezers -20°C<br>Samples were transported on ice to the hospital and immediately stored at -20°C	RT PCR DNA<br>Primers were designed based on 16srRNA<br>FISH and flow cytometric analysis
Shadid et al. [46]	The mothers collected stool samples and were asked to deep-freeze the samples within 20 min<br>The samples were transported in a frozen state and stored at −20 °C	RT PCR DNA<br>Primers were designed based on 16srRNA
Simpson et al. [38]	Sterile sample tubes were used to collect breast milk<br>The women did not receive instructions regarding washing or sterilization of the breast surface<br>Samples were frozen in their home freezer until transportation to the laboratory where they were stored at −80°C	RT PCR DNA<br>Primers were designed based on 16srRNA
Sordillo et al. [23]	Mothers were asked to collect half a teaspoon of their infant's stool from a dirty nappy using a tongue depressor and store in their home freezer<br>These were then stored in the clinic at −80° C	RT PCR DNA<br>Primers were designed based on 16srRNA
Wickens et al. [39]	Post-colostrum breastmilk was collected into sterile containers by mothers and stored at −80°C	RT PCR DNA<br>Primers were designed based on 16srRNA
Wickens et al. [40]	Stool samples collected from infants<br>Samples were held in the home freezer until transportation to the research centre for storage at −80° C	RT PCR DNA<br>Primers were designed based on 16srRNA

Supplementary Data

[i]Abbreviations: FISH; Fluorescent in situ hybridization, RT PCR DNA; Real time polymerase chain reaction deoxyribose-nucleic acid, 16srRNA; 16s ribosomal ribonucleic acid
*Methods did not state how the samples were either collected or analysed.
